# The Association between Multidirectional Speed Performance, Dynamic Balance and Chronological Age in Young Soccer Players

**DOI:** 10.3390/jfmk7020041

**Published:** 2022-05-24

**Authors:** Giordano Scinicarelli, Christoph Offerhaus, Boris Feodoroff, Ingo Froböse, Christiane Wilke

**Affiliations:** 1 Institute of Movement Therapy and Movement-Oriented Prevention and Rehabilitation Sciences, German Sport University Cologne, 50933 Cologne, Germany; b.feodoroff@dshs-koeln.de (B.F.); froboese@dshs-koeln.de (I.F.); wilke@dshs-koeln.de (C.W.); 2 Department of Orthopedic Surgery and Sports Traumatology, Witten/Herdecke University, Sana Medical Centre, 50933 Cologne, Germany; christoph.offerhaus@gmx.de

**Keywords:** prevention, youth athletes, screening tests, y-balance test, lower extremity functional test, limb symmetry index, knee stability, change-of-direction speed, postural control

## Abstract

The ability to maintain a stable single-leg balance stance during a fast change of direction movement is a fundamental aspect both for improving sport-specific skills and for prevention strategies. The aim of this cross-sectional study was to investigate the associations between multidirectional speed performance (MDS), dynamic balance performance (DBP), and chronological age in young and uninjured soccer players. In addition, it was examined whether chronological age and balance can predict variance in speed performance. One-hundred forty-six young male soccer players (age range 11–19) performed the y-balance test (YBT) and the lower extremity functional test (LEFT). Descriptive statistics, Pearson correlation, and multiple regression analysis were executed. The analyses were carried out on the further variables: for the DBP, the YBT composite score % (CS dominant leg/CS non-dominant leg) and limb symmetry index % (LSI) were used; for the MDS, the LEFT time in seconds (s) was used. Findings revealed LEFT scores to have a significant association with chronological age (*p* = 0.000), CS dominant (*p* = 0.019) and LSI (*p* = 0.044) of the YBT. In addition, CS dominant and chronological age explained the variance of the LEFT by 44%, regardless of LSI. To conclude, MDS revealed a strong association with DBP of the dominant side but a small association with LSI. In addition, a small association was found between quick LEFT times and older players. Finally, MDS variance can be predicted from DBP of the dominant side and chronological age in young soccer players. The tests used in this study could be useful screening tools for the detection of performance deficits, the implementation of prevention training programs, and the optimization of selection strategies in soccer academies.

## 1. Introduction

### 1.1. Functional Performance in Soccer Players

Functional performance is a crucial component in order to be a successful player and to perform at high levels without injury [1,2]. Functional parameters such as speed and single-leg balance are fundamental for soccer players. [3]. Another important factor to consider is the chronological age [4]. A recent study defined age-related differences in physical abilities as common aspects in elite soccer academies [5]. In youth athletes, speed and balance tests, i.e., lower extremity functional test (LEFT) and y-balance test (YBT) have been extensively used for screening and scouting strategies [6,7,8]. Their results have been associated with an elevated risk of injury to the lower limbs and with a better chance to advance in the elite categories [6,7,8]. In addition, these tests have been commonly used as standard criteria for returning to sport after injury [9].

### 1.2. Speed Performance in Soccer Players

For soccer players, it is essential to prevail over the opponent on the pitch. High speed performance levels may be helpful to succeed in decisive moments of the game, such as winning a sprint to get to the ball first. The multidirectional speed performance (MDS), i.e., the ability to accelerate, decelerate and perform fast changes of direction in the shortest possible time, is one of the major components of the soccer player’s profile [10,11].

Sheppard & Young defined change of direction as a rapid whole-body movement with a change of velocity or direction, with no perceptual and decision-making factors involved [12]. Multi-faceted influencing factors are involved in change of direction performance, in particular technique, straight sprinting speed, anthropometry, and leg muscle qualities such as left-right muscle imbalances [12].

Also, chronological age has been found to have a significant effect on sports motor competencies such as speed performance [13]. In addition, faster change of direction speed times strongly correlated with greater dynamic balance stability in field sport athletes, including soccer players [3]. Contrarily, no significant age-related effects on speed performance were found during linear-sprint tests (10 m, 30 m) in young soccer players [14]. The lower extremity functional test (LEFT) includes both change of direction and linear sprint movements [6,7,8], but it has been rarely used for association studies with chronological age and single-leg dynamic balance.

### 1.3. Balance Performance in Soccer Players

Soccer is considered a dominant single-legged sport, and balance abilities are extremely required while playing [15]. In fact, not only do soccer players need to perform actions and movements as quickly as possible, but they also need further qualities to perform well such as adequate stability, postural control, and dynamic balance of the lower limbs [16,17]. The dynamic balance performance (DBP) is the ability to maintain proper postural control and stability of the knee while standing on one leg and performing a specific action with the other leg, such as passing or shooting the ball [18]. Significant main effects of age have been already found also for the balance performance in previous research [16]. DBP and postural control of the dominant leg revealed a significant positive correlation with age in young soccer players [19]. Also, previous studies showed that adequate balance capacity could lead to faster speed performances among male youth soccer players [16,17,18]. On the other side, dynamic balance stability was not able to differentiate between faster and slower recreational team sport athletes, including soccer players [20].

### 1.4. Aims

Previous research leads to controversial findings and it remains still unclear the association that MDS could display with DBP and chronological age, as measured with the LEFT and YBT. In addition, we still know little about whether and to what extent speed performance can be associated with and predicted by balance performance and chronological age in young soccer players. Therefore, the aim of this cross-sectional study was to investigate the association between MDS (LEFT), DBP (YBT), and chronological age in young and uninjured soccer players. In addition, to observe whether DBP and chronological age could be significant predictors of MDS. The authors of the present study hypothesised that MDS, DBP, and chronological age might be positively associated. In addition, it was hypothesised that DBP and chronological age might be significant predictors of MDS.

## 2. Materials and Methods

### 2.1. Participants

One hundred and forty-six young and uninjured male soccer players from a 3rd division professional German team were included in this cross-sectional study. Anthropometrics data are presented in Table 1. Players were divided into age groups from under-11 (U11) to under-19 (U19), in accordance with the club organization. Players were involved at competitive (U19) and regional levels (from U11 to U17). The training frequency was 2–4 training sessions per week (1–2 h per training session) and 1 match per week (30–90 min per match) for all players. The inclusion criteria were age ranging between 10–19 years and active participation in soccer activity without any restrictions in practices (2–4 × week) or games (1 × week) over the previous 12 months. The exclusion criterion for this study was the presence, over the previous 12 months, of a lower extremity surgery as well as a lower extremity moderate (between 8–28 days of absence) or severe (>28 days of absence) injury [21]. Written informed consent was obtained prior to test participation from all players or relatives and the study was approved by the ethical committee of the German Sport University (GSU) of Cologne (reference number 056/2018). All players were aware of the potential risks and benefits of the study and complied with the design, protocol, and inclusion criteria. No player has been excluded from the study.

### 2.2. Test Procedures

This study was conducted at the German Sport University (GSU) of Cologne. The tests were supervised by two research assistants. The same test order was used for all players, the YBT first and the LEFT second. Players had to wear only sports t-shirts and shorts while performing the tests. The YBT was performed barefoot in an indoor gym facility on a therapeutic mat (FUCHSIUS multi-media GmbH—RehaMatte—München, Germany) while the LEFT was performed with players’ own running shoes on an athletic indoor track. In the beginning, players were measured in kilograms (kg) using a standard scale for weight and measured in metres (m) using a standard tape measure for height. In order to familiarise them with the tests, standardised instructions and demonstrations were provided at the beginning. Before performing the tests, all players executed a standardised warm-up program including ten min of stationary cycling at low intensity (70 watts) and five min of guided, lower extremity joint mobility consisting of flexion/extension, adduction/abduction, and intra/extra rotation exercises for the pelvis, knee, and ankle joints. For the sake of consistency, players were given three practice trials (per leg) before starting three valid attempts (per leg) for the YBT, while one practice trial before starting two valid attempts for the LEFT. Adequate recovery time was allowed between practice trials (30 s) and valid attempts (60 s) for the YBT as well as between valid attempts (120–180 s) for the LEFT. A 5-min break was applied between the YBT and LEFT tests. The test supervisors decided in real-time whether the tests were carried out correctly or not. If no valid attempt was recorded, the player had to be excluded from the analysis; however, all players recorded at least one valid attempt, and, therefore, all players were included in the analysis. Lastly, verbal encouragement and transcription of the scores took place consistently for all players.

#### 2.2.1. Y-Balance Test (YBT)

**Figure 1:**
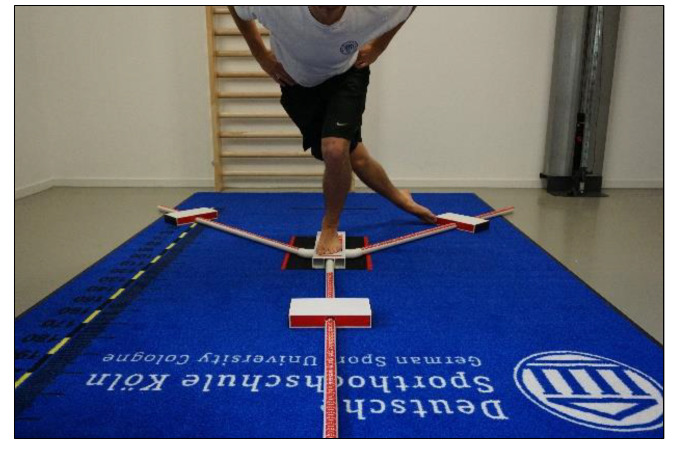
The Y-balance test (YBT).

The YBT (Figure 1) is a reliable and valid test used to measure single-leg DBP, knee stability, and postural control in young populations [22,23,24,25]. The Y-Balance Test Kit (Move2Perform^®^, Evansville, IN, USA) was used for this study. The YBT was performed unilaterally and the leg to be tested first was randomly assigned to avoid learning or fatigue effects. The test was performed in accordance with the standardised procedures proposed in a recent study by Scinicarelli et al. [22]. Players started in a single-leg upright standing position, with the toes of the standing leg at the marked red line of the instrument and the hands fixed on the hip to avoid the influence of arm swing. The sliding elements had to be pushed with the toes of the other leg as far as possible in three given directions: anterior (ANT), posteromedial (PM), and posterolateral (PL). For a correct execution, the standing leg had to maintain a full stance on the platform and the other leg had to maintain constant contact with the sliding elements. The final single-leg balancing position to the starting point had to be maintained for three seconds (s)—measured with a stopwatch—to be considered a valid attempt. Compensatory movements were not allowed, rated as invalid trials, and consequently not included in the data analysis. The following criteria were used to mark invalid attempts: leaving the arms from the hips, inability to maintain balance, touching the ground with the contralateral leg, elevating the heel of the standing leg, kicking the sliding element, or using it as support. Furthermore, limb dominance was determined by the leg with which the player would kick a ball, and used for the calculation of the limb symmetry index (LSI) [26]. The limb length was also measured, as the distance in centimetres (cm) from the greater trochanter to the lateral malleolus, and used for the normalisation of the composite score (CS) [25,27]. Two different performance scores were used: (1) The CS was calculated in percentage (%) using the following formula: [CS = ((ANT + PL + PM)/3 × limb length) × 100] [25]. (2) The CS was used then to calculate the limb symmetry index (LSI) in percentage (%) using the following formula, commonly used for uninjured population: [LSI = (CS non-dominant/CS dominant) × 100] [28,29]. The limb symmetry index (LSI) is usually known as a measure for the level of symmetry in terms of physical or functional performance between the lower limbs [30]. Generally, interlimb differences in performance may occur in single-leg dominant sports such as soccer, regardless of whether or not players have suffered an injury [31]. Therefore, inter-limb symmetry is an adequate investigative method for detecting side-to-side differences in uninjured players. In addition, YBT scores have also been associated with an increased risk of injury to the lower limbs [32], such as CS ≤ 89% [8] and LSI ≤ 90% [6,28,30]. Therefore, the YBT is a useful tool for identifying players with greater DBP and accordingly with a lower risk of injury.

#### 2.2.2. Lower Extremity Functional Test (LEFT)

**Figure 2:**
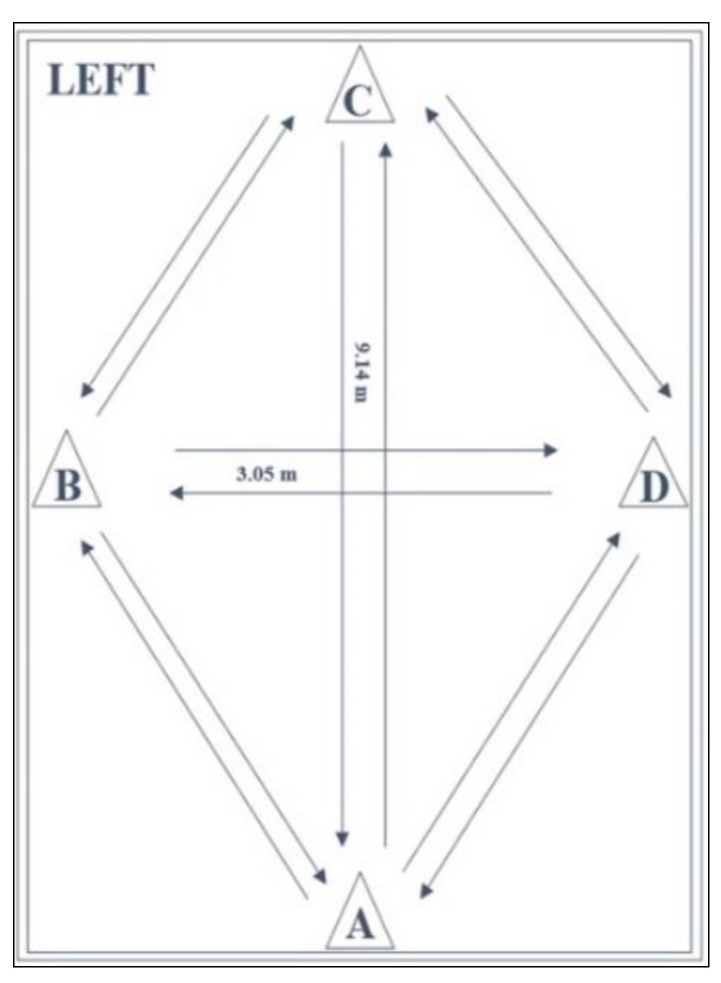
The lower extremity functional test (LEFT) [11,27].

The LEFT is a reliable and valid test for the measurement of MDS, athletic fitness, and fatigue resistance by performing a series of 16 specific manoeuvres as fast as possible, including forward and backward sprinting, sidestepping, cross-stepping, 45° and 90° cutting [7,22,24,33]. The LEFT has been chosen because it requires minimal equipment, is quick to perform, and has been demonstrated to assess athletic fitness and return to sport readiness in youth soccer players [7,22,24,33]. The list of the 16 specific manoeuvres to be performed respect the guidelines provided by Brumitt et al. [7]. The layout (Figure 2) is a combination of four cones in a diamond-shape (9.14 m × 3.05 m). The movement sequence of Figure 2 is explained as follows: (1) Forward sprint (ACA), (2) Backward sprint (ACA), (3) Side shuffle right—face in (ADCBA), (4) Side shuffle left—face in (ABCDA), (5) Cariocas right—face in (ADCBA), (6) Cariocas left—face in (ABCDA), (7) Figure of 8 right—face in (ADCBA), (8) Figure of 8 left—face in (ABCDA), (9) 45° Cuts right (ADCBA), (10) 45° Cuts left (ABCDA), (11) 90° Cuts right (ADBA), (12) 90° Cuts left (ABDA), (13) 90° Crossover cuts right (ADBA), (14) 90° Crossover cuts left (ABDA), (15) Forward sprint (ACA), (16) Backward sprint (ACA). The test was performed in accordance with the standardised procedures proposed in a recent study by Scinicarelli et al. [22]. Players started in an upright standing position with both feet behind the starting point at cone A. On the command of one of the test supervisors, the players performed eight different agility tasks, with each task being performed twice (once to the right and once to the left direction). Because of the multidirectional requirements of the test and the variety of tasks to be performed, verbal instruction of subsequent movements was provided throughout the test. Attempts were considered invalid if participants failed to perform the designated manoeuvres or dropped a cone by contact. Time was measured in seconds (s) using a stopwatch by each of the test supervisors, from the first sprint after the starting signal as soon as players left cone A, to the last sprint as soon as players passed cone A. The final score was calculated by using the mean time in seconds (s) between both stopwatches used by the two supervisors [7,22,24]. The LEFT is considered a soccer-specific measurement, by including both linear- and multidirectional- speed parameters. Such specific actions are performed during matches or training on a regular basis, e.g., forward sprint and backward sprint [24,34]. Furthermore, scores obtained through this test have been also associated with a higher risk of injury to the lower limbs, such as speed time (s) ≤ 100 s [7]. Therefore, the LEFT is a useful test to identify players with higher speed performances and eventually with a higher risk of injury.

### 2.3. Statistical Analysis

SPSS for Windows (Version 26.0, IBM^®^ Corporation, Endicott, NY, USA) was used for all statistical analyses with a significance level set at *p* < 0.05. The normality of data was evaluated by Shapiro–Wilk Test, Skewness (range ± 2), and Kurtosis (range ± 7). Descriptive statistics of anthropometrics, as well as performance scores, were calculated by means and standard deviations (±SD). Outliers were not identified. For each test, the best score among the valid attempts was used for the data analysis. Thus, the following variables were used for the analysis: CS dominant leg (%), CS non-dominant leg (%), and LSI (%) for the YBT (i.e., DBP); execution time (s) for the LEFT (i.e., MDS). Pearson correlation analysis was used to detect the magnitude and statistical significance of the associations between MDS and DBP (dominant/non-dominant leg), interlimb balance symmetry (LSI), and chronological age. The magnitude of the association was set as follows: small 0.1 < r < 0.3; moderate 0.3 < r < 0.5; strong 0.5 < r < 1.0. Multiple regression analysis was run to predict the overall variance of MDS and the relative contribution of each of the predictors: DBP (dominant leg), interlimb balance symmetry (LSI), and chronological age. All required assumptions for the analysis were met by our data: 1, continuous scale dependent variable (MDS); 2, continuous scale independent variables (DBP and chronological age), 3, independence of residuals; 4, assumed linear relationships between variables; 5, homoscedasticity of data; 6, no multicollinearity of data; 7, no significant outliers; 8, residuals approximately normally distributed. The forward selection method was used and after fitting the regression model, the residual plots were checked to avoid biased estimates.

## 3. Results

### 3.1. Descriptive Statistics

The results (mean ± SD) of the functional performance tests are provided by age groups in Table 2. Levene’s test revealed the equality of variances for both dominant (*p* = 0.420) and non-dominant (*p* = 0.584) leg for the DBP (YBT). Tukey’s test revealed homogeneous distribution (*p* > 0.05) for the interlimb balance symmetry (YBT, LSI) and MDS (LEFT). In total (U11-U19), players performed the tests achieving the following results: CS dominant leg (83.8 ± 5.9%), CS non-dominant leg (83.8 ± 5.2%), and LSI (100.1 ± 5.6%) for the YBT; execution time (98.3 ± 6.5 s) for the LEFT. The cut-offs for performance deficits or injury risk indicators for the YBT and LEFT tests previously referred to in the literature are reported at the bottom of Table 2 [7,8,25,30]. All age groups reached poor composite scores in the YBT, for both dominant/non-dominant legs. More than half of the age groups achieved results that are correlated to injury risk in the LEFT (U14-U19).

### 3.2. Association Analysis between Functional Performance Variables and Chronological Age

A Pearson correlation analysis was run to analyse the associations of MDS with DBP (dominant/non-dominant leg and LSI) and chronological age. Results are reported in Table 3. Three out of four variables have been found to be statistically associated with MDS (*p* < 0.05), except for the DBP on the non-dominant leg (*p* > 0.05). MDS showed a strong negative association (*p* < 0.001; r = −0.626) with chronological age: i.e., as years of age increased, multidirectional speed decreased in terms of time (s) and thereby produced a faster execution. MDS showed a small negative association with DBP on the dominant leg (*p* = 0.019; r = −0.194) and interlimb balance symmetry (*p* = 0.044; r = −0.167): i.e., as dynamic balance on the dominant leg (CS, %) and interlimb balance symmetry (LSI, %) increased, multidirectional speed decreased in terms of time (s) and thereby produced a faster execution. Furthermore, no association was found between DBP and chronological age (*p* > 0.05).

### 3.3. Multiple Regression Analysis between Functional Performance Variables and Chronological Age

A multiple regression analysis was run to predict MDS from DBP (CS, dominant leg), interlimb balance symmetry (LSI), and chronological age. Results are reported in Table 4. The model summary shows that these variables statistically predicted overall MDS (F, df 3, 142 = 38.312, *p* < 0.001, R^2^ = 0.477), with a good level of prediction (R = 0.669). In addition, two out of three variables added statistical significance to the prediction (*p* < 0.05), except for the interlimb balance symmetry (*p* > 0.05). From the R Square coefficient, it is assumed that all the predictors explain 44.7% of the variability of the MDS. The formula to predict MDS from DBP and chronological age, can be defined as follows: MDS = 171.8 − [2.7 (s) × years] − [0.4 (s) × CS dominant leg (%)]. This formula is obtained from the relative contribution of each of the predictors contained under the section unstandardised coefficients in Table 4.

## 4. Discussion

The present study explored the associations between MDS, DPB, and chronological age in young and uninjured soccer players. In addition, it was investigated whether MDS can be predicted by DBP and chronological age. The main finding of this study suggests MDS to be significantly associated with single-leg DBP (CS, dominant side), interlimb balance symmetry (LSI), and chronological age. Additionally, single-leg DBP (CS, dominant side) and chronological age were found to be good predictors of MDS variance, regardless of interlimb balance symmetry (LSI). It can therefore be summarised that faster players correspond to the older players and, also, that MDS variance can be predicted by chronological age and DBP of the dominant leg.

### 4.1. Association between Speed and Balance

In the present study, it was assumed that faster MDS may correspond to greater single-leg DBP (CS) and greater limb symmetry index (LSI). Our results confirmed our hypothesis. In fact, greater single-leg balance performance (CS, dominant side: r = −0.194; *p* = 0.019) and interlimb balance symmetry (LSI: r = −0.167; *p* = 0.44) were found to have a small negative association with faster MDS for the soccer players in question. Unfortunately, association studies between balance performance, interlimb asymmetries, and speed performance lead to inconsistent findings [31]. In addition, to the best of the author’s knowledge, there are almost no previous studies investigating the association between dynamic balance and MDS as measured in this study with the YBT and LEFT tests. Therefore, comparison with previous research remains a challenge, and more association studies are needed in order to investigate functional performance in young populations more thoroughly. However, one study showed the YBT to be significantly correlated with change of direction speed performance in young elite soccer players [35] Another study showed moderate associations between DBP (YBT) and speed performance (10 m and 30 m) in prepubescent soccer players [10]. In addition, it was demonstrated that balance training on both stable and unstable surfaces can lead to improvements in linear speed performance (40-Yard-sprint) [36]. Finally, the authors affirm that the activities requiring explosive power, such as change of direction movement, may reflect the ability to managing a better-balanced posture during fast actions [37]. In fact, in order to deal with previously developed musculoskeletal asymmetries and possibly reduce injury risk, older players with longer professional careers seem to stabilize their own knees and ankle joints more appropriately while executing fast movements [34]. Thus, in agreement with previous literature, it can be confirmed also by this study that enhancements in balance performance are associated with enhancements in speed performance.

### 4.2. Association between Speed and Age

For the present study, it was assumed that faster MDS may correspond to higher chronological age. The results of the present study confirmed our hypothesis and revealed a strong negative association (r = −0.626; *p* = 0.000) between the two variables, i.e., decreases in speed time (s) while ageing and therefore a faster MDS for older players. Although Ateş et al. found no association between age and speed performance during linear sprint tests (10 m and 30 m) in young soccer players [14], our finding is in line with the majority of previous studies. In fact, change-of-direction and sprint velocities were found to be positively associated with age at amateur level [38]. In addition, when linear sprint and multidirectional speed tests were executed together, their performances were found to be positively associated with age at elite level [39]. Thus, it can be deduced that speed performance is positively associated with age in young soccer players. Consequently, a particular investigative focus would therefore be essential when dealing with functional tests in young players in relation to long-term career goals. For example, this study shows that a higher speed performance can be expected from older players rather than younger players. Therefore, even though a recent study found that the 30 m sprint test was the best predictor of selection into an elite level youth football academy [40], the authors of the present study suggest that speed performance should not be taken as a unique benchmark for scouting strategies in young players. In fact, speed performance outcomes could be influenced by other factors. According to Valente-dos-Santos et al., skeletal maturity status explains inter-individual variability in maximal short-term run performances with and without the ball possession at early ages of participation in competitive soccer [41]. In addition, Bishop et al. reported that more “stable athletes” can run faster in multidirectional speed tests because of their better force distribution during these actions [31].

### 4.3. Speed Performance Prediction

It has been demonstrated that not only chronological age but also the DBP profile, i.e., single-leg scores and interlimb symmetry, could be positively associated with speed performance. To this concern, for the present study, it was assumed that the variance of MDS could be predicted by single-leg DBP and chronological age. Our results confirmed our hypothesis. In fact, chronological age and DBP on the dominant leg, but not limb symmetry index (LSI), were found to be good predictors (r = 0.669; *p* = 0.001) of the MDS variance. Our independent variables (chronological age and DBP) predicted the variance of our dependent variable (MDS) by 44.7% (r square = 0.447). Specifically, for each additional year of age, there was a reduction in MDS of 2.7 s (*p* < 0.001), while for each additional percentage in the CS of the dominant leg (YBT), there was a reduction in MDS of 0.4 s (*p* = 0.002). Our findings are in accordance with previous literature. In fact, the Y-balance test explained 68% of the variance of change of direction performance in elite soccer players [42]. In another study, it showed age to be a significant predictor of soccer-specific skills, such as change-of-direction speed performance [43]. However, our prediction rate of 44% is low and it should be interpreted with caution. In fact, speed and sprint performance have also been shown to be significantly associated with other influencing factors in youth academy soccer players, such as biological maturation, relative age-effect, training experience, and explosive strength [44,45,46]. However, these factors were not investigated in this study and further research is required.

### 4.4. Future Perspectives

A young athlete with a faster speed performance and a better unilateral balance, could provide more guarantees in terms of injury risk and functional performance, which could also be reflected in better skills in football practice and competition. Therefore, a better understanding of functional performance trends based on associations with chronological age could support sports clinicians in optimising pre-injury screening, scouting strategies, return to sport decision-making, and the implementation of preventive training programs. With regard to our findings, it is worth noting that poor results in single-leg balance performance (CS dom/non-dom ≤ 89%) are offset by good interlimb balance symmetry (LSI ≥ 90%) in all players. Additionally, in relation to the MDS, players from U14 to U19 showed faster execution time (≤100 s), while players from U11 to U13 showed slower execution time (≥100 s) in the LEFT. Nevertheless, if we reason that the cut-offs for YBT and LEFT (Table 1: CS ≤ 89%, LSI ≤ 90%, LEFT ≤ 100 s) have been already associated with a high risk of lower limb injury in young athletes [7,8,25,30], it may be deduced that these results could be a potential injury risk indicator for the players in question. Although injury data were not collected in this study, screening tests should be performed systematically in young and uninjured soccer players, at least twice a season, to identify subjects with greater injury risk indicators for the lower extremities and act preventively with individual training programs. Preventive single-leg balance training, as well as multidirectional speed training, might be the right solution to be integrated at an earlier age (from the u-11) in football academies.

### 4.5. Limitations

This study also has some limitations. First, the influence of height, weight, and BMI were not considered. Second, the effects of biological age and growth process were not evaluated. Also, other maturity-associated variables were not considered as change-of-direction speed determinants. Third, no follow-up nor longitudinal observation for injury documentation were carried out, and thus, no injury risk association can be made. Further studies are required to broader investigate these aspects.

## 5. Conclusions

The present study shows that faster players may correspond to older players in a professional soccer academy. Also, MDS can be predicted by chronological age and DBP of the dominant leg. MDS revealed a strong association with single-leg DBP of the dominant side and a small association with interlimb balance symmetry and chronological age. In addition, single-leg DBP (dominant side) and chronological age explained the variance of MDS by 44%, regardless of interlimb balance symmetry. Furthermore, our participants exhibited single-leg dynamic balance deficits (CS dominant, CS non-dominant ≤ 89%) but good limb symmetry index (LSI ≥ 90%) in all age groups. Therefore, the tests used in this study could be useful screening tools in soccer academies for the implementation of individual and prevention training programs. Specifically, these tests could support talent identification strategies and help sports therapists to detect players with performance deficits or more likely to get injured.

## Figures and Tables

**Table 1:** Anthropometric data (Mean ± SD).

Age Groups (N)	*Mass* *(kg ± SD)*	*Height* *(m ± SD)*	*BMI* *(kg/m^2^ ± SD)*	*Limb Length* *Dom/N-Dom* (*cm± SD)*
**U11 (15)** ***95% CI*** ***COV (%)***	37.2 ± 5.8 ±0.9 13.6%	1.4 ± 0.5 N.A. 4.1%	18.4 ± 2.5 ±0.2 8.0%	81.4 ± 5.4/81.8 ± 5.5 ±0.8/±0.8 5.4%/5.5%
**U12 (18)** ***95% CI*** ***COV (%)***	39.1 ± 4.8 ±0.6 9.8%	1.5 ± 0.5 N.A. 3.4%	18.9 ± 2.3 ±0.2 6.6%	81.2 ± 4.4/81.6 ± 3.3 ±0.5/±0.5 4.1%/4.0%
**U13 (19)** ***95% CI*** ***COV (%)***	49.5 ± 9.6 ±1.3 17.5%	1.6 ± 0.5 N.A. 5.9%	19.7 ± 3.2 ±0.3 11.2	89.3 ± 7.1/89.2 ± 6.9 ±0.9/±0.9 6.8%/6.8%
**U14 (19)** ***95% CI*** ***COV (%)***	57.6 ± 9.3 ±0.3 9.1%	1.7 ± 0.5 N.A. 5.2%	21.2 ± 2.9 ±1.3 14.6%	95.3 ± 5.8/95.5 ± 6.2 ±0.7/±0.8 5.0%/5.4%
**U15 (19)** ***95% CI*** ***COV (%)***	64.4 ± 9.8 ±1.5 15.3%	1.7 ± 0.5 N.A. 4.4%	21.8 ± 2.9 ±0.3 9.0%	97.4 ± 5.7/97.5 ± 5.8 ±0.7/±0.7 4.8%/4.9%
**U16 (21)** ***95% CI*** ***COV (%)***	68.9 ± 9.9 ±1.3 8.7%	1.8 ± 0.5 N.A. 4.7%	22.8 ± 2.9 ±0.3 8.7%	98.3 ± 7.5/98.5 ± 7.6 ±0.9/±1.0 6.6%/6.6%
**U17 (18)** ***95% CI*** ***COV (%)***	72.3 ± 8.5 ±1.2 10.4%	1.8 ± 0.5 N.A. 3.1%	23.8 ± 2.7 ±0.3 7.0%	99.5 ± 5.2/99.5 ± 5.1 ±0.6/±0.6 4.1%/4.1%
**U19 (17)** ***95% CI*** ***COV (%)***	73.9 ± 9.9 ±0.4 9.8%	1.8 ± 0.5 N.A. 3.9%	23.3 ± 3.3 ±0.4 9.8%	101.2 ± 6.5/100.1 ± 6.7 ±0.9/±0.9 5.4%/5.6%
**Total (146)** ***95% CI*** ***COV (%)***	57.9 ± 8.5 ±0.9 11.8%	1.8 ± 0.5 N.A. 4.3%	21.2 ± 2.8 ±0.4 9.4%	92.9 ± 5.9/92.9 ± 5.9 ±0.8/±0.8 5.3%/5.4%

**Abbreviations:** U, under; N, number of players; BMI, body mass index; Dom, dominant leg; N-Dom, non-dominant leg; SD, standard deviation; CI, confidence interval; COV, coefficient of variation; N.A., not available (≅ 0.0); kg, kilograms; m, meters; m^2^, square meters; cm, centimeters.

**Table 2:** Descriptive statistics of the functional performance tests (Mean ± SD).

Age Groups	*Dynamic Balance* *(YBT)*		*Multidirectional Speed* *(LEFT)*
	*Composite Score* *(CS, %)*	*Limb Symmetry Index* *(LSI, %)*	*Execution Time* *(s)*
**U11** **Dom** **N-Dom**	84.0 ± 8.1 82.8 ± 6.7	101.8 ± 10.5	111.1 ± 15.7
**U12** **Dom** **N-Dom**	86.3 ± 5.2 88.8 ± 5.1	97.3 ± 6.8	102.4 ± 3.4
**U13** **Dom** **N-Dom**	84.0 ± 8.2 84.1 ± 5.7	99.8 ± 6.1	107.1 ± 8.4
**U14** **Dom** **N-Dom**	82.2 ± 4.6 83.2 ± 4.1	98.9 ± 3.0	98.6 ± 4.4
**U15** **Dom** **N-Dom**	83.5 ± 5.5 81.9 ± 5.8	102.1 ± 4.3	93.0 ± 4.2
**U16** **Dom** **N-Dom**	83.0 ± 4.9 83.9 ± 4.8	98.9 ± 3.9	92.4 ± 5.0
**U17** **Dom** **N-Dom**	83.3 ± 5.6 82.0 ± 4.7	101.7 ± 5.3	89.9 ± 3.6
**U19** **Dom** **N-Dom**	84.0 ± 5.0 83.9 ± 4.4	100.2 ± 4.5	92.0 ± 7.3

**Abbreviations:** U, under; Dom, dominant leg; N-Dom, non-dominant leg; YBT, y-balance test; LEFT, lower extremity functional test; CS, composite score (%); LSI, limb symmetry index (%); s, seconds; %, percentage. Cut-off for performance deficits of injury risk indicators: CS ≤ 89%, LSI ≤ 90%, LEFT ≤ 100 s.

**Table 3:** Association analysis.

Pearson Correlation between Variables								
	DBP						Chronological Age	
	*Dominant Leg* *(CS, %)*		*Non-Dominant Leg* *(CS, %)*		*Interlimb Symmetry* *(LSI, %)*		*(Years)*	
**MDS** **(Time, S)**	*r*	*p-value*	*R*	*p-value*	*R*	*p-value*	*r*	*p-value*
	−0.194	0.019 *	−0.060	0.469	−0.167	0.044 *	−0.626	0.000 *
**Chronological Age (Years)**	−0.068	0.416	−0.138	0.096	0.057	0.491		

**Abbreviations:** DBP, dynamic balance performance; MDS, multidirectional speed performance; LSI, limb symmetry index; CS, composite score; %, percent; s, seconds; r, Pearson coefficient. *, association is significant (2-tailed) at the 0.05 level.

**Table 4:** Multiple regression analysis.

**Model Summary**							
	*R*	*R Square*	*Adjusted R Square*	*Std. Error of the Estimate*			
**MDS**	0.669	0.447	0.436	7.5173			
**ANOVA—MDS**							
	*Sum of square*	*df*	*Mean Square*	*F*	*Sig.*		
**Regression**	6494.960	3	2164.987	38.312	<0.001 *		
**Residual**	8024.288	142	58.509				
**Total**	14519.248	145					
**Coefficients—MDS**							
*Predictors*	*Unstandardized Coefficients*		*Standardized Coefficients*		*95.0% CI for B*		
	*B*	*Std. Error*	*B*	*T*	*Sig.*	*Lower Bound*	*Upper Bound*
**Constant**	171.823	11.978		14.345	<0.001 *	148.144	195.501
**Chronological Age**	−2.649	0.260	−0.640	−10.178	<0.001 *	−3.164	−2.135
**DBP**	−0.385	0.122	−0.229	−3.162	0.002 *	−0.626	−0.144
**Interlimb Balance Symmetry**	−0.027	0.124	−0.015	−0.214	0.831	−0.272	0.219

**Abbreviations**: DBP, dynamic balance performance; MDS, multidirectional speed performance; R, multiple correlation coefficient; df, degrees of freedom; F, F-ratio; Sig., significant; B, Beta; t, t-value; Std., standard; CI, confidence interval. *, multiple regression is significant at the 0.05 level.

## Data Availability

The data presented in this study are available on request from the corresponding author. The data are not publicly available due to privacy restrictions.
